# Impute.me: An Open-Source, Non-profit Tool for Using Data From Direct-to-Consumer Genetic Testing to Calculate and Interpret Polygenic Risk Scores

**DOI:** 10.3389/fgene.2020.00578

**Published:** 2020-06-30

**Authors:** Lasse Folkersen, Oliver Pain, Andrés Ingason, Thomas Werge, Cathryn M. Lewis, Jehannine Austin

**Affiliations:** ^1^Institute of Biological Psychiatry, Mental Health Centre Sankt Hans, Copenhagen, Denmark; ^2^Social, Genetic and Developmental Psychiatry Centre, Institute of Psychiatry, Psychology & Neuroscience, King’s College London, London, United Kingdom; ^3^Department of Medical & Molecular Genetics, Faculty of Life Sciences & Medicine, King’s College London, London, United Kingdom; ^4^Department of Psychiatry, University of British Columbia, Vancouver, BC, Canada; ^5^Department of Medical Genetics, University of British Columbia, Vancouver, BC, Canada

**Keywords:** genetics, polygenic risk scores, direct-to-consumer, personal genomes, risk prediction

## Abstract

To date, interpretation of genomic information has focused on single variants conferring disease risk, but most disorders of major public concern have a polygenic architecture. Polygenic risk scores (PRSs) give a single measure of disease liability by summarizing disease risk across hundreds of thousands of genetic variants. They can be calculated in any genome-wide genotype data-source, using a prediction model based on genome-wide summary statistics from external studies. As genome-wide association studies increase in power, the predictive ability for disease risk will also increase. Although PRSs are unlikely ever to be fully diagnostic, they may give valuable medical information for risk stratification, prognosis, or treatment response prediction. Public engagement is therefore becoming important on the potential use and acceptability of PRSs. However, the current public perception of genetics is that it provides “yes/no” answers about the presence/absence of a condition, or the potential for developing a condition, which in not the case for common, complex disorders with polygenic architecture. Meanwhile, unregulated third-party applications are being developed to satisfy consumer demand for information on the impact of lower-risk variants on common diseases that are highly polygenic. Often, applications report results from single-nucleotide polymorphisms (SNPs) and disregard effect size, which is highly inappropriate for common, complex disorders where everybody carries risk variants. Tools are therefore needed to communicate our understanding of genetic vulnerability as a continuous trait, where a genetic liability confers risk for disease. Impute.me is one such tool, whose focus is on education and information on common, complex disorders with polygenetic architecture. Its research-focused open-source website allows users to upload consumer genetics data to obtain PRSs, with results reported on a population-level normal distribution. Diseases can only be browsed by *International Classification of Diseases*, 10th Revision (ICD-10) chapter–location or alphabetically, thus prompting the user to consider genetic risk scores in a medical context of relevance to the individual. Here, we present an overview of the implementation of the impute.me site, along with analysis of typical usage patterns, which may advance public perception of genomic risk and precision medicine.

## Introduction

In clinical genetics, testing for rare strong-effect causal variants is routinely performed in the health-care system to confirm a diagnosis or to evaluate individual risk suspected from anamnestic information (Baig et al., 2016), and in such instances, the use of genome sequencing is expanding (Byrjalsen et al., 2018). Meanwhile, outside of the health-care system, direct-to-consumer (DTC) genetics expands rapidly, providing the public with access to individual genetic data profiles and to interpretation of common genetic variants derived from genotyping microarrays (Kaye, 2008; Greshake et al., 2014). This is developing as a sprawling industry of consumer services with widely diverging standards, including third-party genome analysis services. These services typically provide individual results from analysis of common single-nucleotide polymorphisms (SNPs) with (at best) weak effects. They are therefore severely mis-aligned with current state-of-the-art, which at least for common, complex disease is to use polygenic risk scores (PRSs) to estimate the combined risk of common variation in the genome (Lee et al., 2008; Lewis and Vassos, 2017).

**FIGURE 1:**
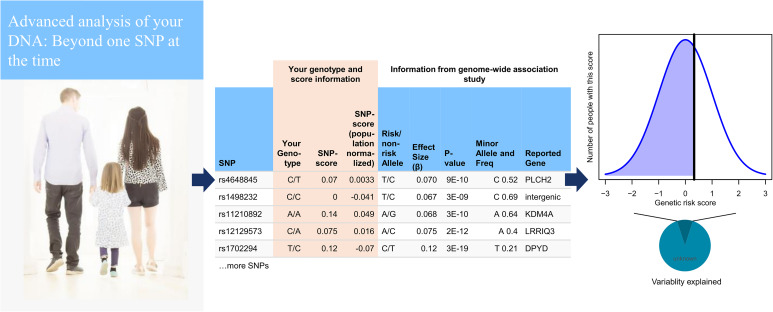
Basic pipeline setup from the user point of view. On upload of a genome, data are checked according quality control (QC) parameters that have been developed to handle most types of microarray-based consumer genetics data. The genome is then imputed using 1000 Genomes as reference (*left*). The imputed data are then further subjected to automated analysis scripts from 15 different modules, most of which are based on polygenic risk score calculations. The calculations include 1,859 traits from genome-wide association studies (GWASs) and 634 traits from the UK Biobank, as well as customized modules for height, and drug response. Most polygenic risk scores use GWAS significant single-nucleotide polymorphisms (SNPs) out of necessity, although 20 major diseases are based on LDpred all-SNP scores (*center*). A user can then browse their scores in relation to the population, shown together with a chart displaying how much variability is explained (*right*).

We believe that the goal of the academic genetics community should extend beyond theory. This means engaging with the public and assisting those who seek information, even when it means helping them to interpret their own genomic data. We therefore developed impute.me as an online web-app for analysis and education in personal genetic analysis. The web-app is illustrated in Figure 1. Using any major DTC vendor, a user can download their raw data and then upload it at impute.me. Uploaded files are checked and formatted according to procedures that have been developed to handle most types of microarray-based consumer genetics data, including an imputation step. These data are then further subjected to automated analysis scripts including PRS calculations. This includes more than 2,000 traits, browsable in different interface types (modules). Each module is designed with the goal of putting findings in as relevant a context as possible, prompting users to see common variant genetics as a support tool rather than a diagnosis finder. The aim is to provide information as broadly as possible to offer a real alternative to the widespread practice of reporting on weak SNP genotypes for any trait, even though that means generation of reports that are below any sensible threshold for clinical usability. We hope that having this as an open and accessible resource for everyone will be of help to the debate on what exactly constitutes clinical usability beyond high-risk pathogenic variants.

In this article, we will describe the (i) development and setup, (ii) validation and testing, (iii) evaluation of usage, (iv) communication of risk scores, and (v) ethics and implications. In the section *Development and Setup*, we discuss some of the challenges faced when developing a full personal-genome scoring pipeline. The goal of this section is to motivate and explain the choices made in development. In the second section, *Validation and Testing*, we use public Biobank data from individuals who consented for genetic research to test the effect of the impute.me scores on known disease outcomes. The purpose of this section is to test and validate scores, as well as to investigate consequences of some of the challenges that were raised in the first section. In the third section, *Evaluation of Usage*, we evaluate usage metrics of impute.me users. The goal of this section is to shed light on behavioral patterns of individuals who use DTC genetics for health questions and to offer recommendations that may be of use in other personal-genome scoring pipelines. In the section *Communication of Risk Scores*, we discuss our views on future directions particularly with respect to improving how genetic findings are presented to people. Finally, in *Ethics and Implications*, we discuss the ethics of providing access to health-related interpretation of DNA data.

## Development and Setup

The first challenge in development of personal genomic services is standardization. As the name impute.me implies, all genotype data are processed by imputation of genotype data (Howie et al., 2009; Delaneau et al., 2013). This procedure expands the data available into ungenotyped SNPs and increases overlap with public genome-wide association study (GWAS) summary statistics used to estimate risk. It also expands the SNP overlap between microarray types from the major vendors, such as 23andMe, MyHeritage, and Ancestry.com. Further, we have found that imputation helps in avoiding major errors, for example, strand-flip issues that arise from the dozens of different data formats. Eliminating such problems from further processing is one important step to minimize mis-interpretation of genome analysis. To ensure high standard of reported results, impute.me requires a fully completed imputation for continued analysis.

The second challenge is to estimate PRSs that are accurate and robust to heterogenous data sources. This is particularly important to an application utilized by people from around the world leveraging data from dozens of different vendors and data types. Importantly, PRSs calculated from GWAS of a population of (for example) European ancestry will perform better for individuals of the same ancestry, and the systematic shift (i.e., bias) in risk scores in individuals from other populations is a problem (Curtis, 2018). Because studies of all disease traits are not yet available for all non-European populations, the pragmatic solution has been to include a population-specific normalization attempting to minimize the systematic shifts of scores for non-European ancestry users. Further, it is computationally and logistically easier to implement PRSs that use only the most (i.e., genome-wide) significant SNPs (often referred to as top SNPs), but the prediction strength is better when more SNPs are included (all-SNP), which, however, is more sensitive to ancestry biases (Lam et al., 2019). The impute.me pipelines calculate PRSs for each trait or disease on the basis of all-SNP-based PRS calculations if full genome-wide summary statistics are available and processed, and top-SNP-based PRS calculations if not.

**FIGURE 2:**
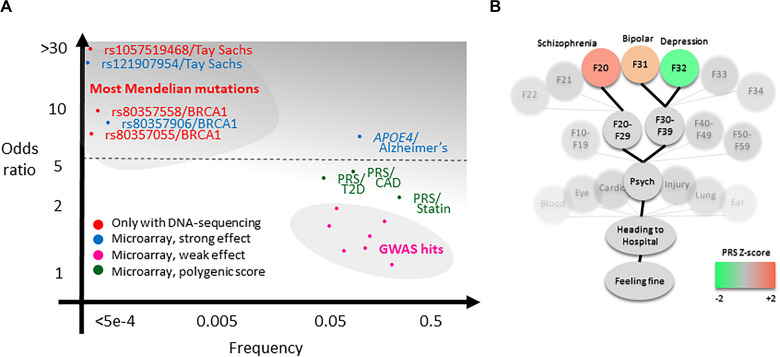
Theoretical background of the analysis pipeline. **(A)** Clinical genetics currently concern high-effect DNA variants that often can only be sequenced (*red*). Additionally, high-effect variants such as APOE4 and a small subset of BRCA1 and BRCA2 pathogenic variants are possible to measure using microarray (*blue* includes several other variants not shown in plot, e.g., Parkinson’s variants). There may be an untapped potential for valuable clinical information in polygenic risk scores (PRSs) for common disease (*green*), for example, for type 2 diabetes (T2D), coronary artery disease (CAD), or statin response (Natarajan et al., 2017; Khera et al., 2018; Wünnemann et al., 2019). It is a primary aim of the impute.me project to make this potential available more broadly, balancing the practice of relying on individual genome-wide association study (GWAS) single-nucleotide polymorphisms (SNPs) and/or reporting of SNP genotypes (*pink*). **(B)** The secondary aim is to provide genetic scores in a relevant context, exemplified in the precision medicine module showing the so-called health-context tree. This tree consists of all entries from the international classification of disease [*International Classification of Diseases*, 10th Revision (ICD-10)], linked to all genetic studies. It allows browsing of PRSs in a relevant context. In the example shown, the tree is open on the psychiatry chapter, showing PRSs for schizophrenia (F20), unipolar depression (F32), and bipolar depression (F31). Although these scores have little predictive relevance for a healthy individual, they may be useful in the context of psychiatric evaluation, particularly in the case of more extreme scores.

The third challenge is presentation. For a single rare large-effect variant, such as for the pathogenic variants in the *BRCA* genes conferring very high risk of cancers (odds ratio >10; Figure 2A, upper left), presentation focuses on absence versus presence (Maxwell et al., 2016). However, also, low-effect variants, for example, as in pharmacogenetics, impacting statin response, is considered as having potential clinical use (Natarajan et al., 2017; Figure 2A, lower right). This difference in effect magnitude is a major challenge in result presentation and understanding, particularly because a firm threshold is difficult to set: In the context of a drug-prescription situation or a question of which of two suspected disease risks is the most likely, it may be useful to know such scores. But in the context of an otherwise healthy individual, genetic risks are only relevant if we are very certain of them, they are serious, and preferably actionable [e.g., BRCA variants (Kalia et al., 2017)]. For this reason, we have made the design choice to avoid the use of lists sorted by risk score. Currently, scores are accessible through either an alphabetically sorted list or in a tree-like setup where genetic scores are reported in a health-context tree (Figure 2B). In this, all scores are included, but scores that are less relevant to healthy individuals (i.e., most of them) are buried deeper into the health-context tree. As further discussed in the section *Future Challenges*, there are a lot of remaining challenges to solve in this question.

## Validation and Testing

To evaluate pipelines on individuals with known disease outcomes, we investigated 242 samples from the CommonMind data set. The CommonMind data set includes patients with schizophrenia (SCZ), bipolar disorder, and controls, from European ancestry and from African ancestry. For each disorder and each ancestry group, the full impute.me pipelines were applied, including imputation and PRS calculation. Additionally, SNP sets corresponding to each of three major DTC companies were extracted and re-calculated. This was done to test the hypothesis that PRS calculation in mixed SNP sets poses particular challenges with regard to missing SNPs. Such sets of genotyped SNPs that are different in each sample are an unavoidable consequence of working with online data uploads.

We found that disease prediction strength, measured as variability explained, corresponded well to theoretical expectations of known SNP heritability (Lee et al., 2017; Li et al., 2017; Wünnemann et al., 2019). Secondly, we found that using all-SNP scores resulted in better prediction than top-SNP scores, which was as expected (Vilhjálmsson et al., 2015). Thirdly, we found that prediction was more accurate in individuals of European ancestry compared with individuals of African ancestry, which is concordant with the PRSs being developed from European Ancestry GWAS (Schizophrenia Working Group of the Psychiatric Genomics Consortium, 2014; Hou et al., 2016; Lee et al., 2018). These observations match well with findings from studies of PRSs in much larger data sets. We caution that universally valid estimates of variability explained are better derived from larger studies that can consider the numerous issues such as balancing of cases and controls, realistic sampling conditions, and other inflations of effects. The intention here is to provide a specific test of impute-me pipelines and address DTC data-related questions.

**FIGURE 3:**
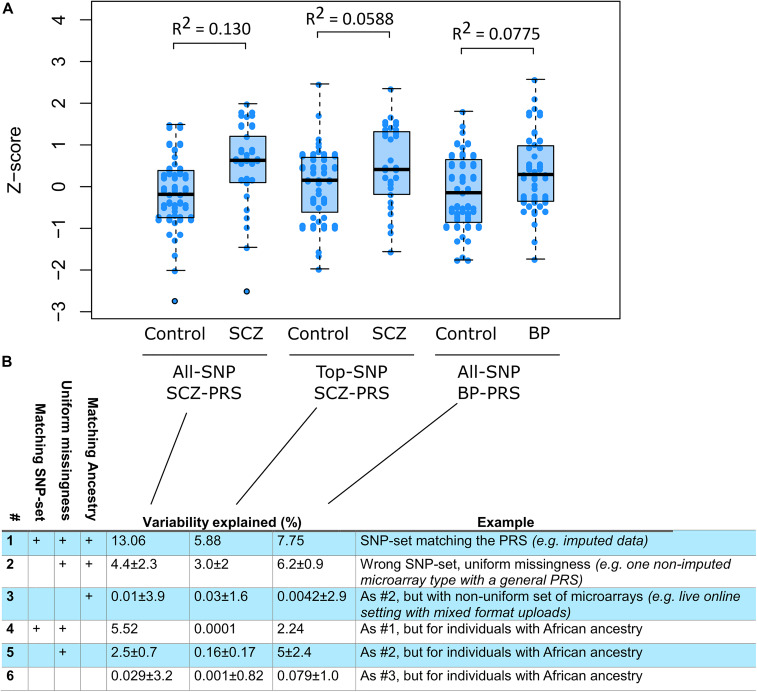
Pipeline evaluation using publicly available genotyped cohorts. **(A)** Three scores were calculated in individuals of European ancestry and relevant diagnoses (*n*_control_ = 39, *n*_SCZ_ = 25, and *n*_BP_ = 39): a schizophrenia (SCZ) all-single-nucleotide polymorphism (SNP) score (*n*_SNP_ = 558,406), an SCZ top-SNP score (*n*_SNP_ = 93), and a bipolar (BP) all-SNP score (*n*_SNP_ = 554977). The BP top-SNP score only used five genome-wide significant SNPs and was not tested. The proportion of variance explained (Nagelkerke *R*^2^) is shown above each case–control pair. **(B)** Testing different conditions of ancestry and input SNP sets. Row #1 corresponds to the variability explained after processing through the full impute.me pipeline, that is, the same calculation shown in the plot. Row #2 shows the prediction level when the polygenic risk score (PRS) algorithm uses input samples from only one type of direct-to-consumer (DTC) vendor, but the algorithm has not been trained specifically for that SNP set. Values are given as mean ± SD of three analyses in which SNP sets were all from 23andMe (v4), ancestry-com, or MyHeritage. row #3 shows the prediction when each sample uses different SNP sets, that is, the actual situation when dealing with user-uploaded DTC data online. Values are given as mean ± SD over 100 random drawings of combinations of the 23andMe (v4), Ancestry.com, and MyHeritage sets, in proportions of 55, 30, and 15%, respectively. These proportions correspond to what are observed in live users. Rows #4–6 shows the same as #1–3 but calculated for CommonMind individuals of African ancestry (*n*_control_ = 47, *n*_SCZ_ = 39, and *n*_BP_ = 6). The corresponding AUC values for this figure are 0.693, 0.614, and 0.634 for row #1: for row #2, 0.55 ± 0.12, 0.53 ± 0.084, and 0.62 ± 0.012; and for row #3, 0.58 ± 0.047, 0.55 ± 0.03, and 0.57 ± 0.047. Additionally, an extended version of the figure is available at www.impute.me/prsExplainer, where additional metrics of prediction can be explored interactively.

Of importance to this, we found that PRS prediction in mixed samples of non-imputed data causes severe problems. When training PRS algorithms, an SNP set is prespecified. The pipelines evaluated here were trained with HapMap3 as SNP set. Similar choices are made in other published PRSs. However, such SNP sets may not match with the SNPs available in downloadable raw data from DTC vendors. We therefore tested what prediction strength would be possible when using raw data directly from DTC vendors, both in a uniform setting (e.g., “all individuals use 23andMe v4 data”) and in a mixed setting (e.g., “individuals have data from different vendors”). We found that in the uniform setting, roughly half the predictive strength remained when using genotype data that are not imputed to match the HapMap3 SNP sets (Figure 3, rows 2 and 4). In the mixed setting, virtually no predictive strength remained (Figure 3, rows 3 and 6). The mixed setting is the reality that is faced, both for third-party analytical services and for DTC vendors with different chip versions. Imputation is therefore likely to be an essential requirement in such scenarios.

To compare these findings with approaches that look at one SNP at the time, we extracted the SNPedia/Promethease SNPs that were indicated as associated with SCZ (Cariaso and Lennon, 2012). All cases (*n* = 25) and all controls (*n* = 39) had at least one risk variant from at least one of the 139 SNPs that indicated SCZ association. When focusing on SNPs that had the SNPedia/Promethease-defined *“magnitude”-*level (*sic*.) at >1.5, we found that 80% of the SCZ cases (20 of 25) had at least one SNPedia/Promethease risk variant. Among the healthy controls, 84% (33 of 39) had at least one such risk variant (*p* = 0.9 for difference in proportions). In other words, it is not very predictive to know if you have a SCZ SNP. This illustrates the importance of considering more than one SNP at the time.

Finally, we compared pipeline reproducibility using two genome-data files, one obtained from MyHeritage and one from Ancestry.com, but sampled from the same person. After processing through the impute-me pipelines, the correlation between PRS values over 1,468 traits was *r* = 0.933 between the two samples. Traits that showed discrepancy between the two data files typically were based on only few SNPs, of which one did not meet imputation quality thresholds for one of the data files.

## Evaluation of Usage

**FIGURE 4:**
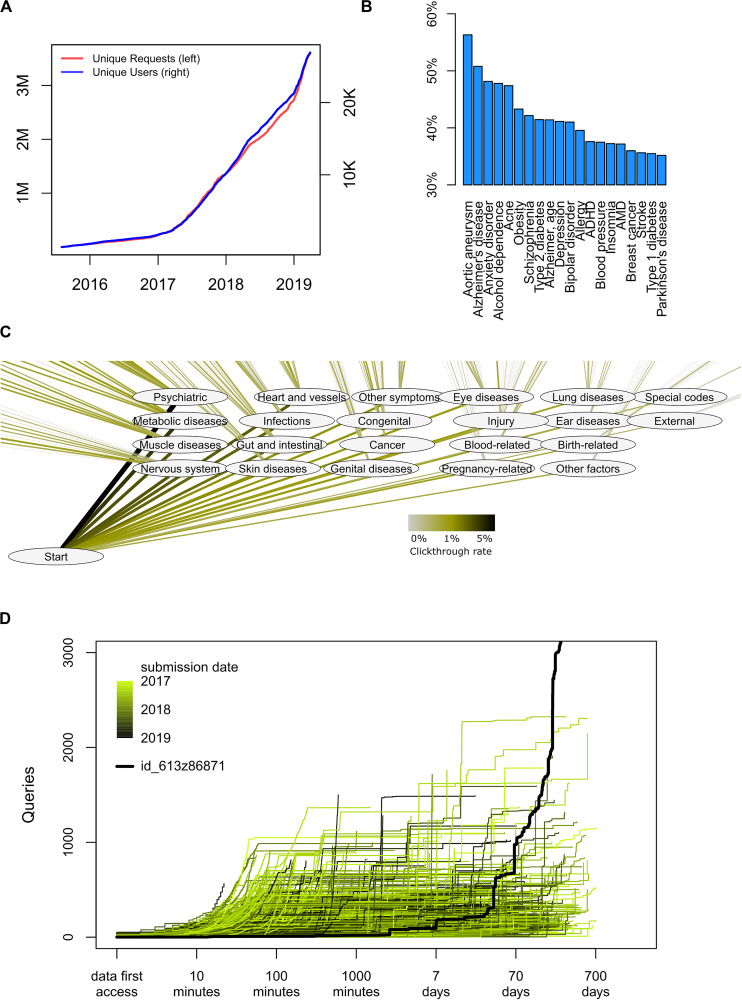
Detailed usage statistics. **(A)** Overall count of unique users and unique analysis requests since August 2015. Each *request* corresponds to a specific analysis, for example, the risk score for a disease, or a view in the *International Classification of Diseases*, 10th Revision (ICD-10)-based map in the precision medicine module. Each *user* corresponds to an uploaded genome with a unique md5sum. There is no check for twins, altered files, or users with data from separate direct-to-consumer (DTC) companies. **(B)** Distribution of user interests in a trait in the *complex disease module*. In this module, each disease entry is presented on an alphabetically sorted list, with aortic aneurysm being the default value. The percentage indicates how many of the users scrolled down and selected this disease at least once (*n*_clicks_ = 871,855). **(C)** Distribution of interests in a trait in the *precision medicine module*. In this module, each disease entry is presented in the layout of the ICD-10 classification system. The click-through rate reflects how many users pursued information in a given chapter or subchapter, as percentage of total amount of clicks (*n*_clicks_ = 114,039). **(D)** Analysis of how individuals use the interface over time. For each user, the number of queries is shown as a function of time after they first access their data. As all data are automatically deleted after 2 years, no queries extend beyond 730 days. The color code indicates the submission date. The highlighted black line indicates the publically available permanent test user with ID id_613z86871, which is omitted from all other analyses.

As of June 2019, a total of 28,651 genomes had been uploaded to impute.me, and a total of 3.1 million analytical queries had been performed (Figure 4A). The following additional observations about user behavior may be of use to the genetics research community.

Common and well-known diseases are the most sought after. By overall click count and comparing over several different modules, there is no doubt that users are most interested in common disease types; diseases of the brain, heart, and metabolism are more requested. Interface design may of course play important roles in such choices. For example, the choice to serve disease traits as alphabetically sorted lists is likely to artificially inflate interest in, for example, abdominal aneurysm (Figure 4B). However, the larger interest in psychiatry, cardiovascular, and metabolic disorders remains also in the precision medicine module, which is not presented as an alphabetically sorted list (Figure 4C). It is possible that greater scientific interest in PRSs in these fields also drives some of these effects, but we cannot explain why other fields where PRSs are actively discussed, such as cancer, are not attracting more attention.

Likewise, it seems that common disease (“complex disease module”) is more sought after than rare disease (“rare disease module”); 95% of all users visit the first, whereas only 70% visit the second. Again, interface design and project goals probably play a big role in this—the landing page headers says *Beyond one SNP at the time*, and the rare disease module is found in the navigation bar only below seven other module entries. But it may also illustrate a central communication challenge for the field: People are more interested in the genetics of common, complex diseases with small effect sizes (Figure 2A, lower right) but may interpret the results as if they were for rare diseases with large effect sizes (Figure 2A, upper left).

Finally, we have observed that usage of health genetic data surprisingly often is not just a test-and-forget event. When plotting query count as a function of time from first data access, we find an expected pattern of intense browsing the hours and days after first data access (Figure 4D). However, many users revisit their data even months and years after first data access, perhaps implying that results are considered and saved and then revisited at a later time in a different context.

## Communication of Risk Scores

Generation of the PRS data presents one set of challenges, but communicating them to people in such a way as to make it both comprehensible and useful presents another (Lipkus and Hollands, 1999; Naik et al., 2012). We believe that this is a crucial unmet need in current genetics research, because presenting PRS data in a way that is useful requires an understanding of people’s motivations for accessing them in the first place.

To date, studies of PRSs have focused on providing people with PRS information in relation to specific conditions [e.g., cancer (Bancroft et al., 2014, 2015; Smit et al., 2018; Young et al., 2018)] for which participants have an indicated risk and exploring understanding and reactions. No studies have examined what motivates people to seek out and access their own PRSs for common complex conditions, and little is known about how people understand or respond to the data they receive.

Polygenic risk scores information is inherently probabilistic in nature, which is well known to be difficult for people to understand (Hallowell et al., 1998; Smerecnik et al., 2009), and receiving information about genetic risk is not necessarily benign. When people receive genetic test results that they perceive to reflect high risk for a condition, this can have negative impact on outcomes like self-perception and affect, and in the case of receiving high-risk test results for Alzheimer’s disease—can actually impact objective measures of cognitive performance (Wilhelm et al., 2009; Dar-Nimrod et al., 2013; Lineweaver et al., 2014; Lebowitz and Ahn, 2017; Turnwald et al., 2019). Therefore, how information about genetic risk is communicated matters.

The literature suggests that when communicating risk, the most useful and effective strategy is to use absolute risks (Lipkus and Hollands, 1999; Reyna et al., 2009; Naik et al., 2012). In the case of PRSs with modest predictive power, however, this may simply result in restating the population prevalence of a disease for everyone (Janssens, 2019). It is therefore important that the predictive strength is also included in this communication; that is how much the genetic component potentially could alter the absolute risk. The genetic component corresponds to the SNP heritability, and we are therefore exploring how to best include this information (e.g., Figure 1, right). Currently, we have registered the SNP heritability for 294 of the reported traits, available as an experimental option called “plot heritability.” We believe that a main future direction is to experiment and expand on how to best communicate this to people.

It will therefore be useful to have a constant flow of people that are interested in interpreting their genetics and expose them to various modes of presentation. Some could involve statistically advanced concepts, like the area under the receiver operating characteristic curve (AUC) and SNP heritability, but others may take simpler approaches, such as the explanatory jar model pioneered for talking with families about genetics (Peay and Austin, 2011; Austin, 2019). One may even imagine layered models of increasing complexity. This should be followed up with questionnaires probing the level of understanding and general impact on users, something that is possible using the impute.me platform.

## Ethics and Implications

Using genetics to maximize the benefits and minimize the harms to individuals and society requires the effective management of the ethical, legal, and social implications of genetics. Researchers have a responsibility to ensure that the technology and the knowledge developed through genetic research are used responsibly, in light of the bioethical principles of beneficence, non-maleficence, justice, and autonomy (Lázaro-Muñoz et al., 2019). Given that for most complex disorders there is currently a lack of data regarding the harms or benefits of accessing PRS information, the fundamental principle in favor of making PRSs available to the public is that of autonomy—in the context of genetic testing, this refers to “the right of persons to make an informed, independent judgment about whether they wish to be tested and then whether they wish to know the details of the outcome of the testing” (Andrews et al., 1994). Accordingly, currently, DTC users can access health information through portals of DTC providers and through third-party applications (Kalokairinou et al., 2018; Tiller and Lacaze, 2018; Ahmed and Shabani, 2019). The problem is that many popular websites do not communicate high-quality genetic knowledge, in part possibly owing to the lack of engagement by the research communities (Badalato et al., 2017). One solution to this problem is to call for regulation and to ban such sites. Alternatively, as we propose here, it is possible to meet user demands and strive to do so as ethically as possible.

To exemplify this, as researchers, we have a choice in whether to provide access to a state-of-the-art PRS for a disease or not. We know that this PRS does not explain everything about the disease, does not account for all the genetic information, and is not part of today’s clinical guidelines. However, we also know that users are already accessing information about disease through DTC genetics. These users may get their information from flawed assumptions of SNP effect sizes or from commercial platforms with little interest in explaining the limitations of the score. We argue that the choice that maximizes the potential for benefits to individuals is to provide the score and to provide it in a setting that puts its consequence in perspective.

An example of such perspective is that of giving reports by disease score, and not by individual risk variant as is currently the case in most third-party analytics apps. Many people carry the high-risk allele for a common variant, but fewer people have a high PRS, which is the sum of all such risk variants. An example of this is the 84% frequency of SCZ risk variants in healthy users according to SNPedia, as reported above. This means that for those autonomously seeking information on health genetics data, the use of PRSs has the potential to decrease the level of induced worry in people in comparison with the current levels. Similarly, smart interface design can actively steer people toward browsing results by indication, and away from the pervasive practice of reporting the worst genetic scores for any disease first. This too may serve to reduce induced worry, in alignment with the general approach of testing only on indication to limit false-positive rates. Finally, of course, adaptive warnings based on risk levels, including referral to resources such as findageneticcounselor.com, is something we continuously strive to optimize.

## Conclusion

In summary, we present impute.me as a fully operational General Data Protection Regulation (GDPR)-compliant genetic analysis engine covering a very broad range of health-related traits, specifically focusing on optimizing possibilities from microarray-based DNA measurements. The challenges, their solutions, and the curation work behind them are highly relevant today in a setting of highly varying quality in interpretation of personal consumer genetics. In the future, we can expect that PRS predictiveness will increase. This will mean a continued and increasing relevance of the platform, even more so as the number of individuals doing genetic testing increases. With a directed push toward responsible use of genetics, this may even prove to be an overall clinical benefit.

## Methods

### Data Privacy and Security

On data submission, each personal genome is assigned a nine-digit alphanumeric unique identifier (“uniqueID”). This uniqueID is used as login and identifier throughout all downstream processes because it has no information that is personally linkable, as opposed to, for example, an email address. The uniqueID is initially linked to two types of data: those that can be traced back to individual that submitted the genome and those that cannot. Genomic data, filename of submitted data, and email address are of the first type: genomic data because it can be used with software such as *gedmatch* to trace family patterns, filename because it often contains the name of the submitter (e.g., 23andMe data use full name as standard), and email for obvious reasons. Data of the traceable type are deleted 14 days after processing, which is the period in which users are able to download their full imputed data sets. The exception is email addresses, which are not deleted but instead unlinked from the uniqueID and kept elsewhere for the purpose of follow-up studies. Either way, this means that 14 days after processing, there exists nothing on the servers that can link results (designated with a uniqueID) with the person who submitted the data (any of the three traceable data types). Thus, even if the database is leaked or lost, it is not possible to link the data to an actual person. After 2 years, the remaining non-traceable data, for example, the derived calculations, the risk scores, and the genotypes of SNPs of specific interest, are all completely deleted. All ingoing and outgoing data transfers are encrypted using Transport Layer Security (TLS 1.3). All storage is encrypted using the AES-256 standard.

This means that all data are collected for specified, explicit, and legitimate purposes in a transparent manner and kept in a form that permits identification of data subjects for no longer than is necessary for the purposes for which the personal data are processed. We therefore consider that these measures both provide adequate security and privacy protection and are in accordance with the GDPR.

### Preprocessing and Bioinformatics

After submission of data, a comprehensive bioinformatic processing of the genotype data takes place. This is done in order of free computing nodes becoming available, consisting of several support programs; first, a shapeit call is made to phase the data correctly (Delaneau et al., 2013), and then an impute2 call is made with 1000 Genomes version 3 as reference (Howie et al., 2009; 1000 Genomes Project Consortium et al., 2015). Although the pipelines are not guaranteed to handle any format they receive, they currently operate with less than 1% processing failures, meaning uploads that cannot proceed through the full quality control and imputation pipelines. The failures are typically due to file formatting errors, missing chromosomes, or any number of other odd data corruptions that real-world data exchange suffers from.

Several customizations have been made with the goal of minimizing memory footprint and thereby allowing running in a clustered fashion on a series of small cloud computers. This allows for relatively easy scaling of capacity: one simple setup (“hub-only”), where calculations are run on the same computer as the website interface. Another is a hub + node-setup, where a central hub server stores data and shows the website, while a scalable number of node-servers perform all computationally heavy calculations. After preprocessing is finished, two new files are created: a *.gen* file with probabilistic information from imputation calls and a *simple format* file with best guess genotypes, called at a 0.9 impute2 INFO threshold. All further calculations are based on these files. A mail with download links to these two files is returned to the user, along with a JSON-formatted file containing a machine-readable summary of all calculations, as well as links with guidance to obtain more in-depth information on personal DNA interpretation (Folkersen, 2018).

### Polygenic Risk Score Calculation

From the preprocessed data, a modular set of trait predictor algorithms is applied. For many of the modules, the calculations are trivial. For example, this could be the reporting of presence and/or absence of a specific genotype, such as ACTN3 and ACE-gene SNPs known to be (weakly) associated with athletic performance. These are included mostly because users expect them to be. For others, we rely heavily on PRSs.

An important distinguishing factor between different PRS algorithms is how risk alleles are selected. A commonly used approach includes variants based on whether they surpass a given *p*-value threshold in the GWAS, retaining only linkage disequilibrium (LD)-independent variants using LD-based clumping, often with a *p*-value threshold of genome-wide significance (*p* < 5e^–8^). Herein, we refer to this approach as the “top-SNP” approach. The top-SNP approach has the advantage that it is simple to explain, is easy to obtain for many GWAS, and has a light computational burden (e.g., Buniello et al., 2019; Lambert et al., 2019; Patron et al., 2019; Watanabe et al., 2019). However, research has repeatedly shown that the inclusion of variants that do not achieve genome-wide significance improves the variance explained by PRSs, with PRSs including all variants often explaining the most variance. PRSs based on GWAS effect sizes that have undergone shrinkage to account for the LD between variants have been shown to explain more variance than PRSs that account for LD via LD-based clumping (Vilhjálmsson et al., 2015). Herein, we refer to this approach as the all-SNP approach. It is more computationally and practically intensive to implement at scale. Consequently, within impute.me, each trait or disease reported shows all-SNP-based PRS calculations if such is available, and top-SNP-based PRS calculations if not.

In the top-SNP calculation mode, the results are scaled such that the mean of a population is zero and the standard deviation (SD) is 1, according to the relevant 1000 Genomes super-population: African, admixed American, East Asian, European, or South Asian.

$${\mathrm{Population}\text{-}\mathrm{score}}_{\mathrm{snp}}\;=\;{\mathrm{frequency}}_{\mathrm{snp}}\times 2\times {\mathrm{beta}}_{\mathrm{snp}}$$

$$\begin{array}{rcl} \mathrm{Zero}\text{-}\mathrm{centered} & & \;\;\;\;\text{-}\mathrm{score}\;=\\ & & \sum {\mathrm{Beta}}_{\mathrm{snp}}\times {\mathrm{Effect}\text{-}\mathrm{allele}\text{-}\mathrm{count}}_{\mathrm{snp}}\\ & & {\text{-}\mathrm{Population}\text{-}\mathrm{score}}_{\mathrm{snp}} \end{array}$$

$$\begin{array}{rcl} Z\text{-}\mathrm{score}\; & = & \;\mathrm{Zero}\text{-}\mathrm{centered}\text{-}\mathrm{score}/\\ & & {\mathrm{Standard}\text{-}\mathrm{deviation}}_{\mathrm{population}} \end{array}$$

where beta [or log(odds ratio)] is the reported effect size for the SNP effect allele, frequency_SNP_ is the allele frequency for the effect allele, and the Effect-allele-count_SNP_ is the allele count from genotype data (0, 1, or 2).

In the all-SNP calculation, the scaling is similar but done empirically, that is, based on previous impute.me users of matching ethnicity. This mode of scaling is also available as an optional functionality in the top-SNP calculations and generally seems to match well with the default 1000 Genomes super-population scaling.

The all-SNP scores were derived using weightings from the LDpred algorithm (Vilhjálmsson et al., 2015). This algorithm adjusts the effect of each SNP allele for those of other SNP alleles in LD with it and also takes into account the likelihood of a given allele to have a true effect according to a user-defined parameter, which here was taken as *wt1*, that is, the full set of SNPs. The algorithm was directed to use hapmap3 SNPs that had a minor allele frequency >0.05, Hardy–Weinberg equilibrium *p* > 1e^–05^, and genotype yield >0.95, consistent with our expectation that these would be the best imputed SNPs after full pipeline processing.

### Pipeline Testing

To test the pipelines described herein, the CommonMind genotypes measured with the microarray of the type H1M were downloaded along with phenotypic information. Each sample was processed through the impute.me pipelines, using the batch upload functionality. Reported ethnicity was compared with pipeline (genotype) assigned ethnicity and found to be concordant.

After pipeline completion, we extracted three PRSs for each sample, corresponding to SCZ all-SNP, SCZ top-SNP, and BP all-SNP. In the github repository for impute.me, these three correspond to the scores labeled *SCZ_2014_PGC_EXCL_DK.EurUnrel.hapmap3.all.ldpred.effects*, *schizophrenia_25056061*, and *BIP_2016_PGC.All.hapmap3.all. ldpred.effects* trait IDs (Schizophrenia Working Group of the Psychiatric Genomics Consortium, 2014; Hou et al., 2016). These extracted scores formed the basis of the row #1 and #4 calculations in Figure 3. The remaining rows were created by subsetting the best guess imputed genotypes into new sets of users, corresponding to each of three major DTC vendors and then re-running the scoring algorithms with either uniform data or mixed data. Uniform data are here defined as all 195 samples having the same set of SNPs available, corresponding to one of three DTC vendors in each run. Mixed data are defined as samples having different sets of SNPs available, a set corresponding to actual distributions of customers from different DTC vendors, with distributions redrawn 100 times. We estimated the predictive ability of the PRSs using Nagelkerke’s *R*^2^ and AUC.

### Usage Evaluation

A log data freeze was performed on June 8, 2019 by making a copy of all usage log files and then removing the uniqueID of each user. This was done to prevent it from being linked with the genetic data of that user. The exception was the publicly available permanent test user with ID id_613z86871, which was lifted out before analysis and is not included in other summary statistics. Generally, a user corresponds to an uploaded genome with a unique md5sum. Click-through rates were calculated as fraction of users that performed any query in the module in question; for example, the precision medicine module was only launched in September 2018 and, therefore, only counts clicks from people who have used it. Plots were generated using base-R version 3.4.2 and cytoscape version 3.71.

## Urls

Code repository: https://github.com/lassefolkersen/impute-me Web resource: https://www.impute.me/

## Data Availability Statement

Publicly available datasets were analyzed in this study. This data can be found here: CommonMind data doi: 10.7303/syn2759792.

## Ethics Statement

The studies involving genotypes of human participants were reviewed and approved by the CommonMind Consortium. This data is generated from postmortem human brain specimens originating from tissue collections at the Mount Sinai NIH Brain Bank and Tissue Repository, University of Pennsylvania Brain Bank of Psychiatric illnesses and Alzheimer’s Disease Core Center, The University of Pittsburgh NIH NeuroBioBank Brain and Tissue Repository, and the NIMH Human Brain Collection Core. Written informed consent for participation was not required for this study in accordance with the national legislation and the institutional requirements.

## Author Contributions

LF coded the code. All authors contributed to interpretation, drafting the work, critical revision for important intellectual content, and final approval of the manuscript.

## Disclaimer

The views expressed are those of the author(s) and not necessarily those of the NHS, the NIHR or the Department of Health and Social Care.

## Conflict of Interest

The authors declare that the voluntary donations received by impute.me go to a registered company, from where all of it is used to pay for server-costs. The company is a Danish-law IVS company with ID 37918806, financially audited under Danish tax law.
